# Complete Genome Sequence of the *Campylobacter cuniculorum* Type Strain LMG 24588

**DOI:** 10.1128/genomeA.00543-17

**Published:** 2017-06-15

**Authors:** William G. Miller, Emma Yee, Joana Revez, James L. Bono, Mirko Rossi

**Affiliations:** a Produce Safety and Microbiology Research Unit, Agricultural Research Service, U.S. Department of Agriculture, Albany, California, USA; b European Centre for Disease Prevention and Control, Stockholm, Sweden; c Meat Safety and Quality Research Unit, Agricultural Research Service, U.S. Department of Agriculture, Clay Center, Nebraska, USA; d Faculty of Veterinary Medicine, University of Helsinki, Helsinki, Finland

## Abstract

*Campylobacter cuniculorum* is a thermotolerant species isolated from farmed rabbits (*Oryctolagus cuniculus*). Although *C. cuniculorum* is highly prevalent in rabbits farmed for human consumption, the pathogenicity of this organism in humans is still unknown. This study describes the whole-genome sequence of the *C. cuniculorum* type strain LMG 24588 (=CCUG 56289^T^).

## GENOME ANNOUNCEMENT

*Campylobacter cuniculorum* is a thermotolerant species isolated from farmed rabbits (*Oryctolagus cuniculus*) (1, 2). Although rabbit meat is consumed by humans, there have been no reported cases of *C. cuniculorum*-related human illness. The pathogenicity of this organism is unknown; however, similar to *Campylobacter jejuni*, the *C. cuniculorum* type strain was shown to adhere to and invade Vero, HeLa, and COLO205 cells (3). The *C. cuniculorum* type strain LMG 24588 (=150B^T^ = CCUG 56289^T^) was isolated in Italy from the cecal contents of a farmed rabbit (2). In this study, we report the first closed genome sequence of the *C. cuniculorum* type strain.

The Roche GS-FLX, Illumina HiSeq, and PacBio RS next-generation sequencing platforms were used to complete the genome of the *C. cuniculorum* type strain LMG 24588. Shotgun and paired-end Roche 454 reads were assembled (using Newbler version 2.6) into a single array of 200 unique and repeat chromosomal contigs. Most of the contig gaps were closed using Illumina HiSeq reads (SeqWright, Houston, TX) and/or PCR amplification/Sanger sequencing. However, the repeat topography of the LMG 24588^T^ chromosome required PacBio sequencing for both genome closure and assembly validation. Two single-contig plasmid scaffolds were closed/circularized, and all base calls were validated using Illumina HiSeq reads (913× coverage). The final coverage across the genome was 1,363×.

*C. cuniculorum* strain LMG 24588^T^ has a circular genome of 1,931 kbp, with an average G+C content of 31.6%. Protein-, rRNA- and tRNA-coding genes were identified as described previously (4). The genome contains 1,786 putative protein-coding genes, 63 pseudogenes, and 2 sets of rRNA genes. The LMG 24588^T^ chromosome contains six putative genetic islands: one encodes a putative type VI secretion system, and another encodes a putative type IV secretion system. The chromosome also contains 89 G+C tracts ≥8 bp. Seventy-seven of these tracts were demonstrated to be hypervariable; thus, the *C. cuniculorum* type strain is predicted to encode at least 60 contingency genes. Two small plasmids, pCUN1 (4,923 bp) and pCUN2 (1,834 bp), were identified in the LMG 24588^T^ genome.

The Embden-Meyerhof-Parnas glycolytic pathway is incomplete in *Campylobacter* spp.; therefore, these organisms cannot utilize sugars as a carbon source, with the exception of fucose for some strains (5, 6). However, some *C. jejuni* and *Campylobacter coli* strains encode a complete set of enzymes for the alternative Entner-Doudoroff pathway (7–9), which has been shown to be functional (9). Similar to *C. jejuni* and *C. coli*, strain LMG 24588^T^ is also predicted to encode a complete Entner-Doudoroff pathway; however, unlike *C. jejuni* and *C. coli*, this gene cluster is not embedded within an rRNA locus.

Another noteworthy locus contained in *C. cuniculorum* is *tcuABC*, which is involved in tricarballylate utilization (10). Within *Campylobacter* spp., these genes were also identified in the reptile-associated *C. fetus* subsp. *testudinum* and *C. iguaniorum* (11, 12) and the swine-associated *C. hyointestinalis* subsp. *lawsonii* (11). Tricarballylate utilization was proposed to be associated with the hindgut niche potentially inhabited by these organisms (11). Rabbits, like pigs and reptiles, are also hindgut fermenters; thus, tricarballylate utilization may be used by *C. cuniculorum* to more efficiently colonize their hosts.

### Accession number(s).

The complete genome sequence of *C. cuniculorum* strain LMG 24588^T^ has been deposited in GenBank under the accession numbers CP020867 (chromosome) and CP020868 and CP020869 (plasmids pCUN1 and pCUN2, respectively).
